# Controlled delivery of nilotinib using LDH/Fe_2_O_3_-modified chitosan nanocomposite hydrogel beads for the treatment of chronic myeloid leukemia

**DOI:** 10.1038/s41598-026-57760-3

**Published:** 2026-06-10

**Authors:** Milad Gholami, Hori Ghaneialvar, Somayeh Molaei, Naser Abbasi, Ali Aidy, Mahsa Abbasi

**Affiliations:** 1 Biotechnology and Medicinal Plants Research Center, Ilam University of Medical Sciences, P.O. Box 6939177143, Ilam, Iran; 2 Department of Chemistry, Faculty of Science, Kurdistan University, Sanandaj, Iran; 3 Department of Pharmacology, Faculty of Medicine, Ilam University of Medical Sciences, Ilam, Iran; 4 Department of Chemistry, Basic of Science Faculty, Ilam University, Ilam, Iran

**Keywords:** Nilotinib, Drug delivery, Nanocomposite, Hydrogel beads, Chronic myeloid leukemia, Biocompatibility, Biotechnology, Cancer, Chemistry, Drug discovery, Materials science, Nanoscience and technology

## Abstract

Chronic myeloid leukemia remains a significant challenge in cancer treatment because conventional drug delivery systems often fail to provide sustained release and targeted therapeutic effects. To address these limitations, this study a chitosan-based nanocomposite hydrogel system for the controlled delivery of nilotinib, a tyrosine kinase inhibitor used to treat chronic myeloid leukemia. Fe_2_O_3_-containing layered double hydroxide nanocomposites into were incorporated chitosan hydrogels under controlled synthesis conditions (70 ± 2 °C, stirring speed at 800 rpm) to fabricate chitosan nanocomposite hydrogel beads based on LDH and Fe_2_O_3_ nanoparticles. Structural characterization using scanning electron microscopy and X-ray diffraction confirmed successful nanoparticle incorporation and the formation of a porous hydrogel network. Nilotinib was incorporated into the hydrogel beads using both in situ and post-synthetic approaches, and drug release was quantified by high-performance liquid chromatography. Among the formulations investigated, the Post-synthetic incorporation of nilotinib into dried hydrogel beads at a concentration of 20% yielded the highest cumulative nilotinib release after six hours (72.895 ± 0.004 µg/mL). Cytotoxicity studies revealed a concentration-dependent inhibition of chronic myeloid leukemia cell lines (BV173, EM-2, CML-T1, and JOSK-M) and indicated acceptable biocompatibility with human umbilical vein endothelial cells. In addition, the nanocomposite hydrogel demonstrated notable antioxidant activity in the DPPH assay. Overall, the developed LDH/Fe_2_O_3_-modified chitosan hydrogel system represents a promising platform for the controlled delivery of hydrophobic anticancer agents such as nilotinib.

## Introduction

Oxidative stress plays a crucial role in the pathogenesis and progression of various hematological malignancies, including leukemia. An imbalance between reactive oxygen species (ROS) production and cellular antioxidant defenses can lead to DNA damage, genomic instability, and altered signaling pathways, thereby contributing to uncontrolled cell proliferation and resistance to apoptosis^1–4^. Notably, specific types of leukemia, such as chronic lymphocytic leukemia (CLL) and chronic myeloid leukemia (CML), have demonstrated elevated levels of oxidative stress^2,5^. Given the established link between redox balance and leukemogenesis, therapeutic strategies that modulate oxidative stress, alongside conventional treatments such as chemotherapy, are gaining attention. For instance, antioxidant compounds have been investigated for their potential to either directly target leukemic cells by inducing oxidative damage or apoptosis, or to protect healthy cells from chemotherapy-induced toxicity^6–9^.

CML is a hematologic malignancy originating from hematopoietic stem cells due to the Philadelphia chromosome, a translocation between chromosomes 9 and 22. This rearrangement produces the BCR-ABL1 fusion protein, a constitutively active tyrosine kinase that drives leukemogenesis by promoting uncontrolled proliferation and inhibiting apoptosis^10^. Tyrosine kinase inhibitors (TKIs) have significantly improved treatment for CML by selectively targeting BCR-ABL1 activity. Nilotinib, a second-generation TKI, is more potent than Imatinib and effectively manages resistance in patients with ABL kinase domain mutations^5^. However, long-term use is limited by cardiovascular complications, systemic toxicity, and acquired drug resistance, highlighting the need for advanced drug delivery strategies to improve bioavailability and therapeutic efficacy^11^. Layered double hydroxides (LDHs) are a class of anionic clays with the general chemical formula:

$$[(M^{II})_{1-x} (M^{III})_{x}(OH)_2]^{x}+(Anion^{m-}{_{x/n}}) nH_2O]$$

Divalent M^II^ and trivalent M^III^ cations form a positively charged framework intercalated with anions. LDHs offer tunable interlayer spacing, high biocompatibility, and controlled drug release, making them attractive for cancer therapy^12^. Chitosan-based nanocomposite hydrogels (CNH) have emerged as promising drug carriers due to their biodegradability, non-toxicity, and ability to form stable matrices for sustained release^13^. Incorporating nanoparticles (NPs) into CNH further enhances functionality by enabling external control of drug release and targeted therapy^14^. Various NPs, including clay^15^, graphene^16^, hydroxyapatite^17^, and polymeric NPs^18^, have been explored to improve hydrogel-based drug delivery. Yet the integration of LDH/Fe_2_O_3_ within a single chitosan-based hydrogel for CML treatment remains largely unexplored. The primary challenge in CML therapy is eradicating leukemic stem cells, which contribute to relapse. Advanced delivery systems capable of site-specific, sustained delivery are therefore critical to minimize systemic toxicity and enhance therapeutic outcomes. Recent advances in nanomedicine have underscored the importance of oxygen-supplying nanomaterials for overcoming tumor hypoxia and enhancing therapeutic efficacy in cancer treatment^19^. In addition, lysosome-targeted drug delivery systems have demonstrated improved intracellular drug release and therapeutic performance^20^.

In this study, our developed CNH, designed for the controlled delivery of nilotinib, a TKI widely used to treat CML^21^, may offer additional therapeutic benefits through its inherent antioxidant properties. While the primary focus of this study is nilotinib delivery, the potential contribution of the hydrogel matrix itself to the overall therapeutic outcome warrants investigation. Therefore, a preliminary evaluation of its antioxidant capacity was conducted to explore this potential ancillary effect.

## Materials and methods

### Materials

All reagents and chemicals used in this study were of analytical grade and used without further purification. Nilotinib (C_28_H_22_F_3_N_7_O, ≥ 98%, Sigma-Aldrich, USA) was selected as the model TKI. Medium-molecular-weight Chitosan (75–85% deacetylation, MW 50–190 kDa, Sigma-Aldrich, USA) served as the hydrogel matrix. Iron (III) chloride hexahydrate (FeCl_3_·6H_2_O, ≥ 98%, Merck, Germany) was used as the precursor for Fe₂O₃ NPs. Nickel(II) nitrate hexahydrate (Ni(NO₃)₂·6 H₂O, ≥ 98%, Merck, Germany) was used for LDH NP synthesis. Sodium tripolyphosphate (STPP, ≥ 98, Sigma-Aldrich, USA) was employed as a cross-linking agent for Chitosan hydrogel formation. Glacial acetic acid (CH_3_COOH, ≥ 99%, Merck, Germany) was used as the solvent for chitosan dissolution. Sodium hydroxide (NaOH, pellets, ≥ 98%, Merck, Germany) was used to adjust pH during synthesis and gelation. Sodium borohydride (NaBH_4_, ≥ 98%, Sigma-Aldrich, USA) served as a reducing agent during NPs synthesis. Phosphate buffer solution (0.1 M, pH 3.5; Sigma-Aldrich, USA) was used for drug-release experiments. Human CML cell lines (BV173, EM-2, CML-T1, and JOSK-M) and normal human umbilical vein endothelial cells (HUVECs) were obtained from the Pasteur Institute Cell Bank (Tehran, Iran). All cells were cultured in RPMI-1640 medium supplemented with 10% fetal bovine serum (FBS) at 37 °C in a humidified 5% CO_2_ atmosphere.

### Instruments

X-ray diffraction (XRD) patterns were recorded on a Philips PW1730 diffractometer (The Netherlands) using Ni-filtered Cu Kα radiation (λ = 1.542 Å) at 40 kV and 30 mA. Field emission scanning electron microscopy (FE-SEM) images were obtained on a MIRA III microscope (TESCAN, Czech Republic) at 10–20 kV. High-performance liquid chromatography (HPLC) analyses were performed on a KNAUER PLATINblue M-1 HPLC system (KNAUER, Germany) with a UV detector, and a EurospHer II 100-5 C18 analytical column (250 × 4.6 mm, KNAUER) with a guard column. The mobile phase consisted of phosphate buffer (pH 3.5) and acetonitrile at a 30:70 (v/v) ratio, delivered at a flow rate of 1 mL/min. The injection volume was 20 µL, and detection was carried out at 260 nm.

### Synthesis of LDH NPs

For the synthesis of LDH NPs, 0.548 g of Ni(NO₃)₂·6 H₂O (3 mmol) and 0.213 g of Al(NO₃)₃•9 H₂O (1 mmol) were dissolved in 10 mL of deionized water under gentle stirring. The resulting solution was added dropwise (0.5 mL/min) to 40 mL of 0.15 M NaOH while maintaining continuous stirring (600 rpm) at 25 °C ± 1 °C. The pH of the reaction medium was kept constant at 10.5 ± 0.2 throughout the synthesis. After complete addition, the mixture was vortexed for 10 min and then centrifuged at 8000 rpm for 15 min. The precipitate was washed three times with deionized water to remove residual ions and impurities. Finally, the purified LDH NPs were dried in an oven at 100 °C for 16 h to obtain the final LDH NPs^22^.

### Synthesis of Fe_2_O_3_ NPs

For the synthesis of Fe₂O₃ NPs, 50 mL of 0.3 M NaOH was added dropwise (~ 1 mL/min) to 50 mL of 0.1 M FeCl₃·6 H₂O under continuous stirring (600 rpm) at 25 °C ± 1 °C. The resulting mixture was subjected to ultrasonic-assisted precipitation at 80 W and 50 °C for 1 h using an ultrasonic probe. During sonication, the solution color gradually changed from yellow (before sonication) to reddish-brown, confirming the formation of Fe₂O₃ NPs. The precipitate was collected by filtration, washed twice with deionized water to remove residual byproducts, and then calcined at 500 °C for 1 h in air to obtain the thermodynamically stable $\:\alpha\:-$Fe₂O₃ (hematite) phase^23^.

### Synthesis of LDH-Fe_2_O_3_ composites

To synthesize the LDH-Fe_2_O_3_ composite, 0.1 g of Fe_2_O_3_ NPs was added to 50 mL of an LDH NPs suspension and sonicated at 80 W in an ultrasonic bath for 2 h to achieve homogeneous dispersion. After sonication, the composite particles were magnetically separated and oven-dried at 100 °C for 16 h to obtain the LDH-Fe_2_O_3_ composite powder^22^.

### Production of CNH beads based on LDH NPs (CNH-LDH beads)

To prepare CNH-LDH beads, 1 g of medium-molecular-weight chitosan was dissolved in 35 mL of deionized water containing 2.5 mL of acetic acid (added dropwise at approximately 0.5 mL/min) under continuous magnetic stirring (400 rpm) for 1 h. The final chitosan concentration was 2.5% (w/v), ensuring complete dissolution. Because chitosan is soluble only under acidic conditions, acetic acid facilitates dissolution by protonating its amine groups. Subsequently, LDH NP suspensions at concentrations of 5, 10, 15, and 20% (w/v) were incorporated separately into the chitosan solution and stirred for 30 min to ensure homogeneous dispersion. The resulting mixtures were injected dropwise through a 21G syringe into an alkaline crosslinking solution containing 4 g of STPP and 2 g of NaBH₄ under gentle stirring (200 rpm) at 25 °C ± 1 °C. NaBH_4_ acted as a reducing agent, facilitating the crosslinking reaction between STPP and chitosan. The formed beads were left in the crosslinking solution for 24 h at 25 °C ± 1 °C to complete gelation. After crosslinking, the CNH-LDH beads were repeatedly washed with deionized water until the washing solution reached pH 7.0 to remove any residual NaBH₄ and STPP. Finally, the beads were air-dried at 25 °C ± 1 °C under sterile conditions before further characterization^24^.

### Production of CNH beads based on LDH/Fe_2_O_3_ NPs (CNH-LDH@Fe_2_O_3_ beads)

The CNH-LDH@Fe_2_O_3_ beads were prepared using the same procedure as the CNH-LDH beads, except that suspensions containing different weight percentages of the LDH/Fe_2_O_3_ NPs were used. The resulting mixture was injected into the crosslinking solution and allowed to gel for 24 h at 25 °C ± 1 °C. After crosslinking, the beads were thoroughly washed with deionized water to remove residual reagents and air-dried under sterile conditions at room temperature before further characterization.

### Nilotinib incorporation into CNH beads

#### Incorporation of Nilotinib during CNH beads synthesis (Syn-CNH beads)

##### Synthesis of CNH-LDH containing Nilotinib (Syn-CNH-LDH beads)

Syn-CNH-LDH beads were prepared according to the procedure in Sect.  2.6, with the drug added to the chitosan solution. Briefly, 2 mL of 1 mg/mL Nilotinib stock solution (prepared in a water: acetonitrile mixture, 1:9 v/v) was added to a 1 g of chitosan dissolved in 35 mL of deionized water. Subsequently, LDH NP suspensions at concentrations of 5–20% (v/w) were incorporated into the solution, and the mixture was stirred until homogeneous. The subsequent bead formation and cross-linking steps were carried out as described in Sect.  2.6. Nilotinib was selected as a model hydrophobic drug due to its poor aqueous solubility^25^.

##### Synthesis of CNH-LDH@Fe_2_O_3_ beads containing Nilotinib (Syn-CNH-LDH@Fe_2_O_3_ beads)

Syn-CNH-LDH@Fe_2_O_3_ beads were prepared using the same procedure described in Sect.  2.9.1.1. In this formulation, LDH-Fe_2_O_3_ NP suspensions at concentrations of 5–20% (v/w) were used instead of LDH suspensions. The mixture was thoroughly stirred to ensure uniform dispersion of NPs within the chitosan matrix before cross-linking. LDH-based nanocomposites have previously been investigated as potential carriers to improve the delivery of hydrophobic drugs such as nilotinib^25^.

#### Post-synthetic incorporation of Nilotinib into dried hydrogel beads (Dry-CNH beads)

Dry-CNH beads were prepared by immersing dried hydrogel beads based on LDH NPs (Dry-CNH-LDH beads) and LDH-Fe_2_O_3_ NPs (Dry-CNH-LDH@Fe_2_O_3_ beads) separately in a 1 mg/mL nilotinib solution (water: acetonitrile, 1:9 v/v) for 72 h at room temperature. After incorporation, the beads were thoroughly washed with deionized water to remove excess drug and residual solvent, then air-dried under sterile conditions. This post-synthetic approach may reduce the likelihood of drug degradation during bead preparation and facilitate the incorporation surface-associated drugs. Due to its poor aqueous solubility, nilotinib may benefit from nanocomposite-based delivery systems that improve drug dispersibility^25^.

### Drug release from CNH beads

The in vitro release of Nilotinib from CNH-LDH and $\:{\mathrm{CNH-LDH@Fe}}_{2}{\mathrm{O}}_{3}$ beads was evaluated by immersing 1 g of each formulation in 10 mL of phosphate-buffered saline (PBS, $\:\mathrm{pH}=7.2)\:$ at $\:{37}^{\circ\:}\mathrm{C}\text{}$ for 0.5 to 6 h under gentle stirring to simulate physiological conditions.

At predetermined time intervals, 600 µL aliquots were withdrawn and filtered through a 0.22 μm syringe filter. Subsequently, 30 µL of each sample was analyzed by HPLC using a C18 column (250 × 4.6 mm, 5 μm). The HPLC mobile phase consisted of a mixture of phosphate buffer and acetonitrile (30:70, v/v, $\:\mathrm{pH}=3.5)$ at a flow rate of 1 mL/min, with detection at 266 nm. Drug concentrations were determined using a pre-established calibration curve based on peak area and retention time. All release experiments were performed in triplicate, and the results are presented as mean $\:\pm\:$ standard deviation (SD).

### Toxicity assessment of CNH beads

HUVEC cells and CML cell lines, including BV173, EM-2, CML-T1, and JOSK-M were obtained from the Pasteur Institute cell bank (Tehran, Iran).

#### Evaluating the cytotoxicity of CNH beads on HUVEC cells using the Alamar blue assay

HUVEC cells at passage three were cultured in RPMI-1640 medium supplemented with 10% FBS, 2 mM glutamine, streptomycin (100 µg/mL), and penicillin (100 U/mL) at 37 °C under 95% humidity and 5% CO_2_. Once cells reached approximately 80–90% confluence, they were detached with trypsin-EDTA, and cell viability was assessed by the trypan blue exclusion method. A total of 3,000 cells were seeded into each well of a 96-well plate and incubated for 24 h. Subsequently, 100 µL of CNH beads at different concentrations (0, 1, 2, 3, 7, 15, 31, 62, 125, 250, 500, and 1000 µg/mL) was added to the wells. In addition to untreated cells used as the negative control, a drug-free CNH nanocomposite control group was included to evaluate the intrinsic cytotoxicity of the carrier system independently of nilotinib^26^. All treatments were evaluated in triplicate.

After 72 h of incubation, cell viability was evaluated using the Almar Blue assay (Sigma). The Alamar Blue reagent was prepared by dissolving the dye powder in Dulbecco’s Phosphate-Buffered Saline (DPBS) buffer (5.54 mg/50mL), followed by sterilization using a 0.2 μm membrane filter. The prepared solution was stored at low temperature until use. Subsequently, 10 µL of Alamar Blue solution was added to each well, and the plates were incubated for 4 h. Absorbance was measured at 570 nm using a BioTek ELISA reader (ELx 800). Untreated cells served as the control group, while wells containing only culture medium were used as blanks. Cell viability was calculated using the following equation^27^.

$$\%\, Viability = (Mean\,OD\,Sample/Mean\,OD\,Control) \times 100.$$

#### Evaluation of cytotoxicity of CNH beads in CML cell lines using Almar blue assay

The cytotoxic effects of CNH beads were evaluated in CML cell lines, including JOSK-M, EM-2, CML-T1, and BV173, using the Alamar Blue assay method. These cell lines are suspension-type hematological cancer cells that do not require surface attachment for growth.

Following confirmation of biocompatibility in HUVEC cells, the assay was performed to assess the selective cytotoxicity of the prepared formulations against leukemic cells. Briefly, Cells were seeded in 96-well plates at a density of 3000 cells per well. Subsequently, 100 µL of CNH beads at various concentrations (0, 1, 2, 3, 7, 15, 31, 62, 125, 250, 500, and 1000 µg/mL) was added to each well, and the plates were incubated for 72 h under standard cell culture conditions. In addition to untreated cells used as controls, a drug-free CNH nanocomposite control group was included to assess the potetial cytotoxicity of the carrier system.

After incubation, cell viability was assessed using the Alamar Blue assay as described in Sect.  2.8.1. All experiments were performed in triplicate, and the results are presented as mean $\:\pm\:$ standard deviation.

### Evaluation of antioxidant activity using the DPPH assay

The antioxidant activity of CNH-LDH@Fe₂O₃ beads was evaluated using the DPPH radical-scavenging assay. This method is based on the reduction of the stable free radical 2,2-diphenyl-1-picrylhydrazyl (DPPH) by antioxidant compounds, resulting in discoloration of the purple DPPH solution to a yellow-colored reduced form.

Briefly, 2 mL of DPPH solution (100 µM in methanol) was mixed with 2 mL of CNH-LDH@Fe₂O₃ bead suspensions at concentrations of 1, 2, 3, 7, 15, 31, 62, 125, 250, 500, and 1000 µg/mL. The reaction mixtures were incubated in the dark at room temperature for 30 min. Subsequently, absorbance was measured at 520 nm using a SpectraMax Gemini XS microplate reader.

The percentage of DPPH radical-scavenging activity was calculated using the following equation:

$$\:DPPH\:scavenging\:activity\:\left(\%\right)=\left[\right(A_o\:-\:A_1)/A_o]\:\times\:\:100$$

where A₀ represents the absorbance of the control solution, and A₁ represents the absorbance of the sample^28^.

Butylated hydroxytoluene (BHT) was used as the positive control due to its well-established antioxidant, and free-radical-scavenging activity. All measurements were performed in triplicate, and the results are presented as mean ± SD.

### Statistical analysis

All experiments were performed in triplicate, and the results are presented as mean ± SD. Statistical were conducted using SPSS 21.0 software. Significant differences among groups were evaluated using one-way analysis of variance (ANOVA) followed by Duncan’s multiple range test. Differences were considered statistically significant at *p* < 0.05.

## Results

### SEM analyses

**Fig. 1:**
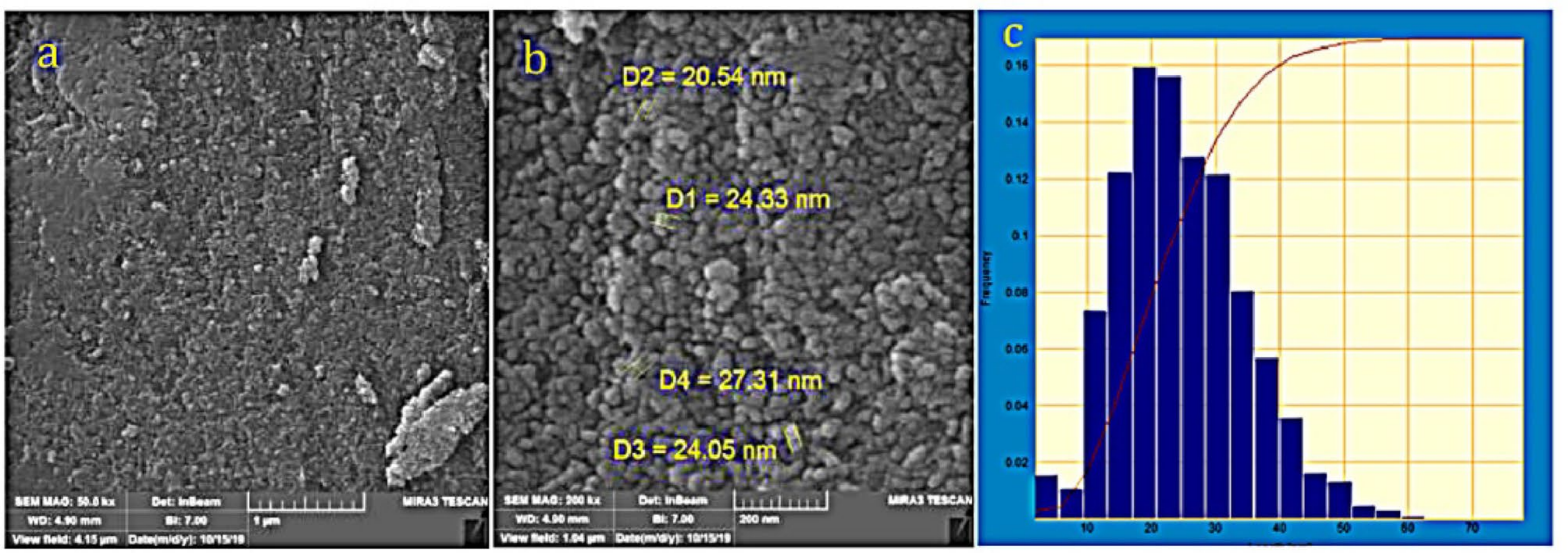
SEM micrographs of a pure LDH sample and its histogram.

**Fig. 2:**
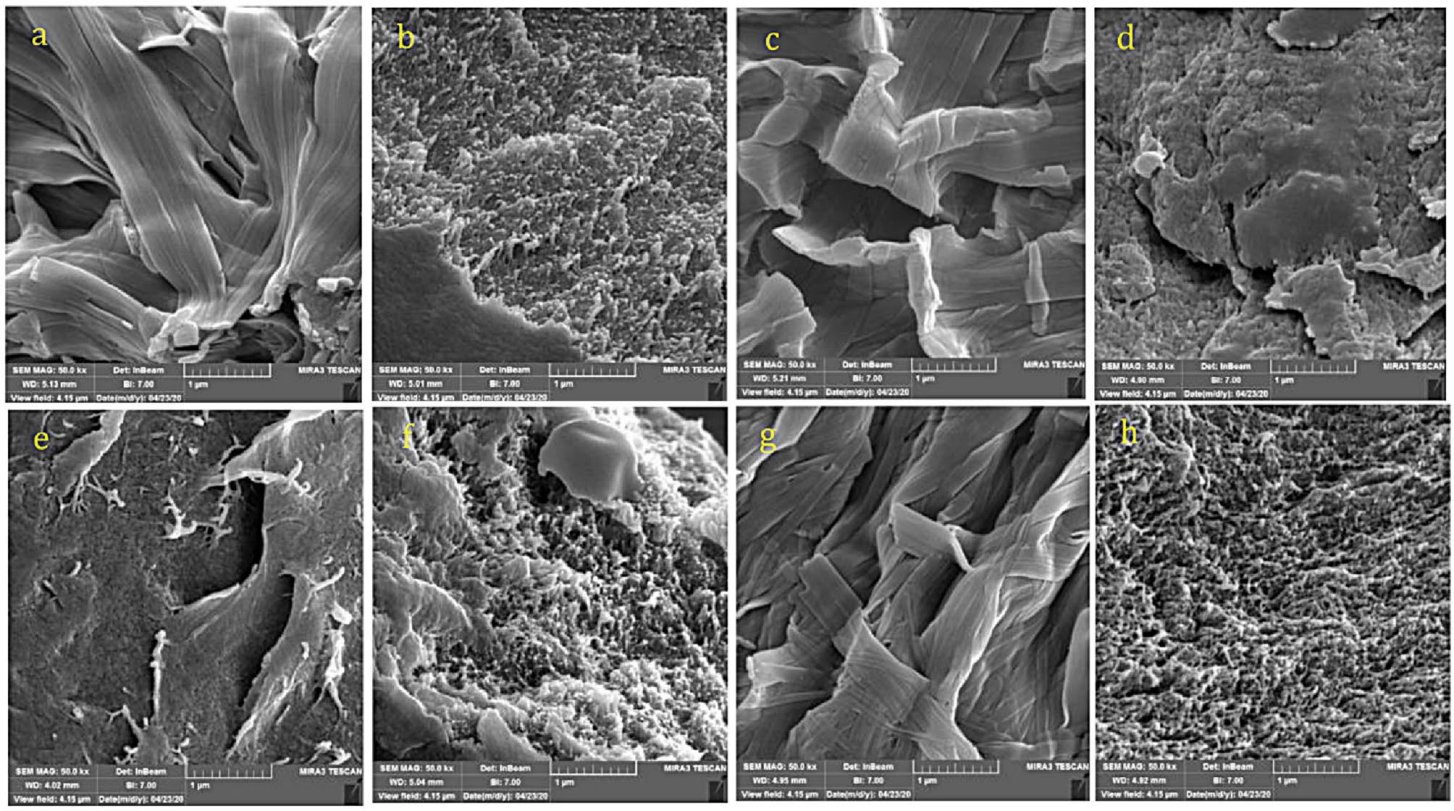
SEM micrographs of Dry-CNH-LDH beads (**a**–**d**) containing 5%, 10%, 15%, and 20% LDH, respectively, and Dry-CNH-LDH@Fe_2_O_3_ beads (**e**–**h**) at the corresponding concentrations.

**Fig. 3:**
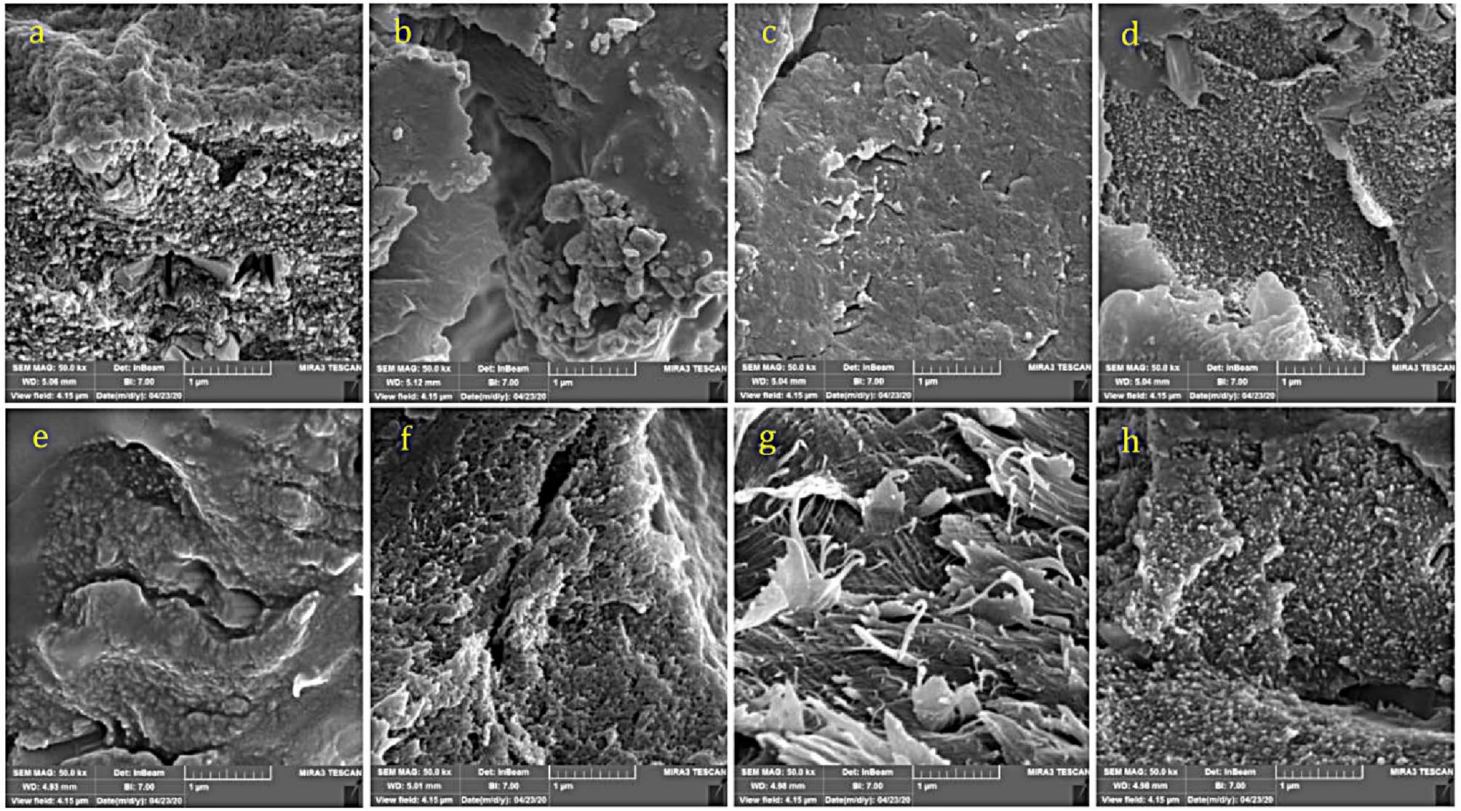
SEM micrographs of Syn-CNH-LDH beads (**a**–**d**) with 5%, 10%, 15%, and 20% LDH; Syn-CNH-LDH@Fe2O3 beads (**e**–**h**) at the same concentrations.

SEM analysis was performed to investigate the surface morphology of Ni-Al-LDH NPs (Fig. 1) and of Dry-CNH-LDH, Dry-CNH-LDH@Fe_2_O_3_, Syn-CNH-LDH, and Syn-CNH-LDH@Fe_2_O_3_ beads at nanoparticle incorporation ratios of 5, 10, 15, and 20 wt% (Figs. 2 and 3). Figure 1 presents SEM images of Ni-Al-LDH NPs at different magnifications, along with the corresponding particle-size distribution histogram. Figures 2 and 3 show SEM micrographs of hydrogel bead formulations prepared using two drug-loading approaches, namely drug incorporation during hydrogel synthesis and post-synthesis drug loading. In each formulation, either LDH NPs alone (Figs. 2a–d and, 3a–d) and, LDH/Fe₂O₃ nanocomposites (Figs. 2e–h and, 3e–h ) were incorporated into the chitosan hydrogel matrix at concentrations of 5, 10, 15, and 20 wt%.

The SEM micrographs revealed rough, wrinkled, and porous surface morphologies characteristic of chitosan-based hydrogel matrices. The observed wrinkles and surface irregularities may be attributed to partial collapse and shrinkage of the hydrogel network during drying. In addition, variations in porosity among formulations may relate to differences in NP loading and the structural organization of the crosslinked hydrogel matrix^29^. The interconnected porous network may facilitate drug encapsulation and diffusion within the hydrogel structure. Dispersed spherical NP domains observed throughout the hydrogel matrix indicate successful incorporation of LDH and LDH/Fe₂O₃ nanocomposites into the hydrogel framework. Similar morphological behavior has previously been reported for nanoparticle-loaded chitosan hydrogels^30^.

Furthermore, increasing the NPs incorporation ratio from 5 to 20 wt% increased the density of spherical NPs domains and produced noticeable changes in surface roughness and porosity, suggesting that NPs loading influences the hydrogel network’s structural organization. The particle-size distribution histogram shown in Fig. 1(c) was obtained by measuring NPs diameters from SEM micrographs using ImageJ software^30^.

### XRD analyses

**Fig. 4:**
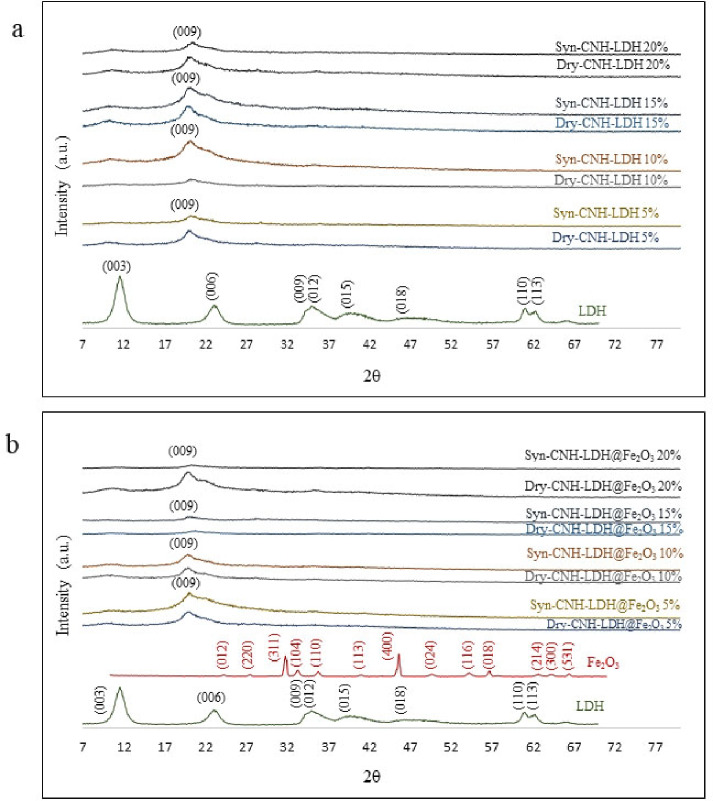
XRD patterns of LDH NPs, Syn-CNH-LDH beads, and Dry-CNH-LDH beads (**a**); XRD patterns of LDH NPs, Fe_2_O_3_ NPs, Dry-CNH-LDH@Fe_2_O_3_ beads, and Syn-CNH-LDH@Fe_2_O_3_ beads (**b**).

Figure 4a presents the XRD patterns of LDH NPs and CNH-LDH@Fe_2_O_3_ beads. The characteristic diffraction peaks of (003), (006), (009), (012), (015), (018), (110), and (113) in pure Ni-Al-LDH correspond to the hexagonal phase of Ni–Al hydrotalcite (JCPDS No. 15–0087), confirming the crystallinity and structural integrity of the synthesized materials^31^. The presence of these peaks in the synthesized nanocomposite beads confirms the successful formation of the LDH crystal structure and the absence of additional crystalline impurities. Figure 4b shows the XRD pattern of Fe₂O₃ NPs, exhibiting distinct peaks at 2θ = 30.1°, 35.5°, 43.1°, 57.2°, and 62.7°, which agree well with the standard Fe₂O₃ phase (JCPDS No. 33–0664)^32^. Broad diffraction peaks centered at 2θ = 22° in all nanocomposite hydrogel samples are attributed to the amorphous nature of the chitosan-based hydrogel.

### HPLC analyses

The in vitro release profile of nilotinib from four hydrogel formulations was evaluated using HPLC, including Syn-CNH-LDH@Fe₂O₃ 20%, Syn-CNH-LDH 20%, Dry-CNH-LDH 20%, and Dry-CNH-LDH@Fe₂O₃ 20%.

**Fig. 5:**
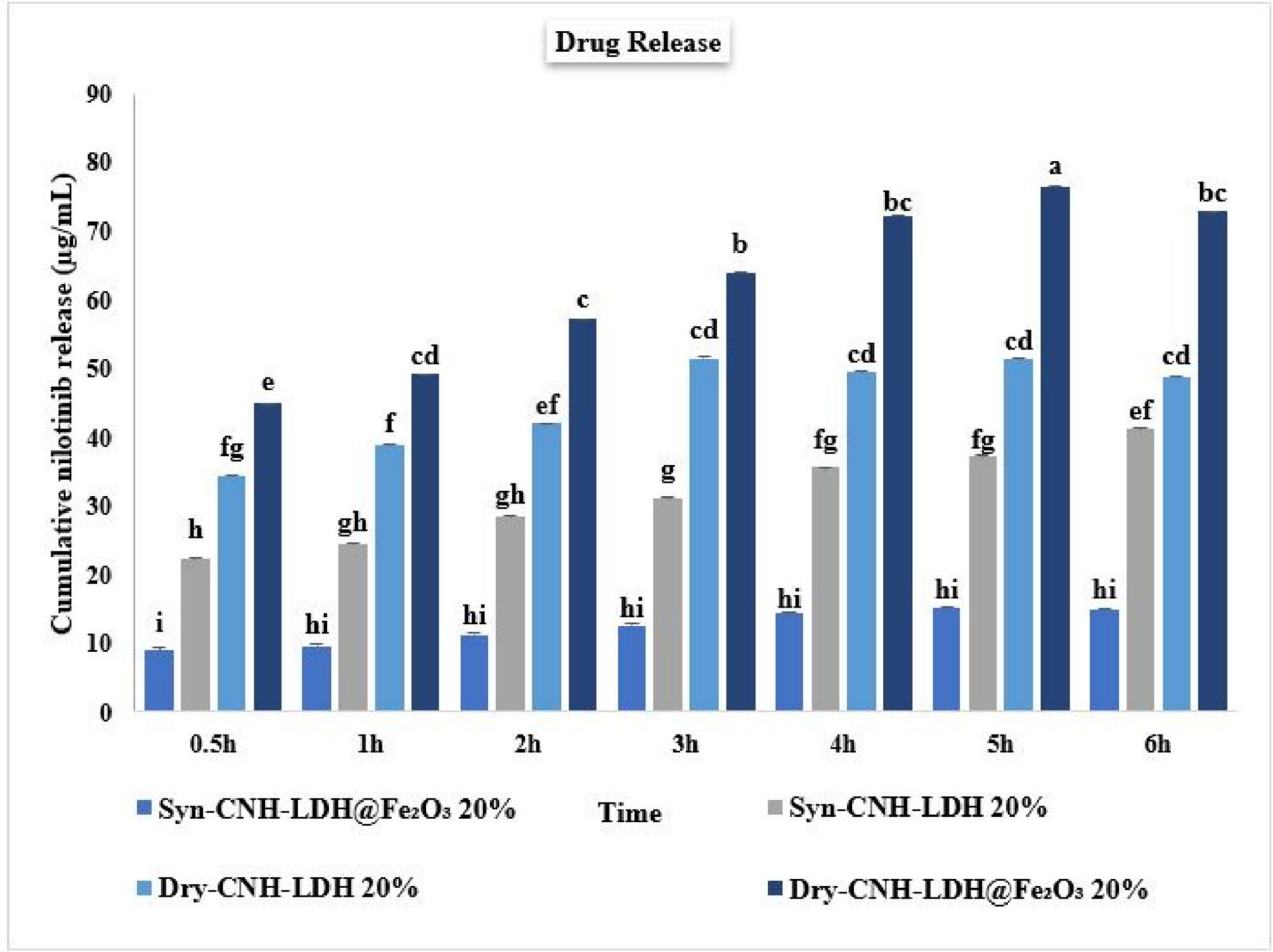
In vitro release profile of nilotinib from different hydrogel bead formulations: Dry-CNH-LDH (20%), Dry-CNH-LDH@Fe_2_O_3_ (20%), Syn-CNH-LDH (20%), and Syn-CNH-LDH@Fe_2_O_3_ (20%).

As shown in Fig. 5, the cumulative released nilotinib concentration (µg/mL) varied among the formulations over the six hours release period. The Dry-CNH-LDH@Fe₂O₃ 20% formulation exhibited the highest cumulative release, reaching (72.895 $\:\pm\:\:$0.004) µg/mL after 6 h (*p* ˂ 0.05), whereas Syn-CNH-LDH@Fe₂O₃ 20% showed the lowest release (14.8$\:\pm\:$0.163 µg/mL).

Differences in release behavior among the formulations may be associated with variations in hydrogel structure, NP incorporation, and drug-loading approach. The release experiments were performed in triplicate, and the results are presented as mean ± SD.

### Cytotoxicity of hydrogel beads on HUVEC and CML cells

#### Cytotoxicity of CNH-LDH@Fe_2_O_3_ beads on HUVEC cells

**Fig. 6:**
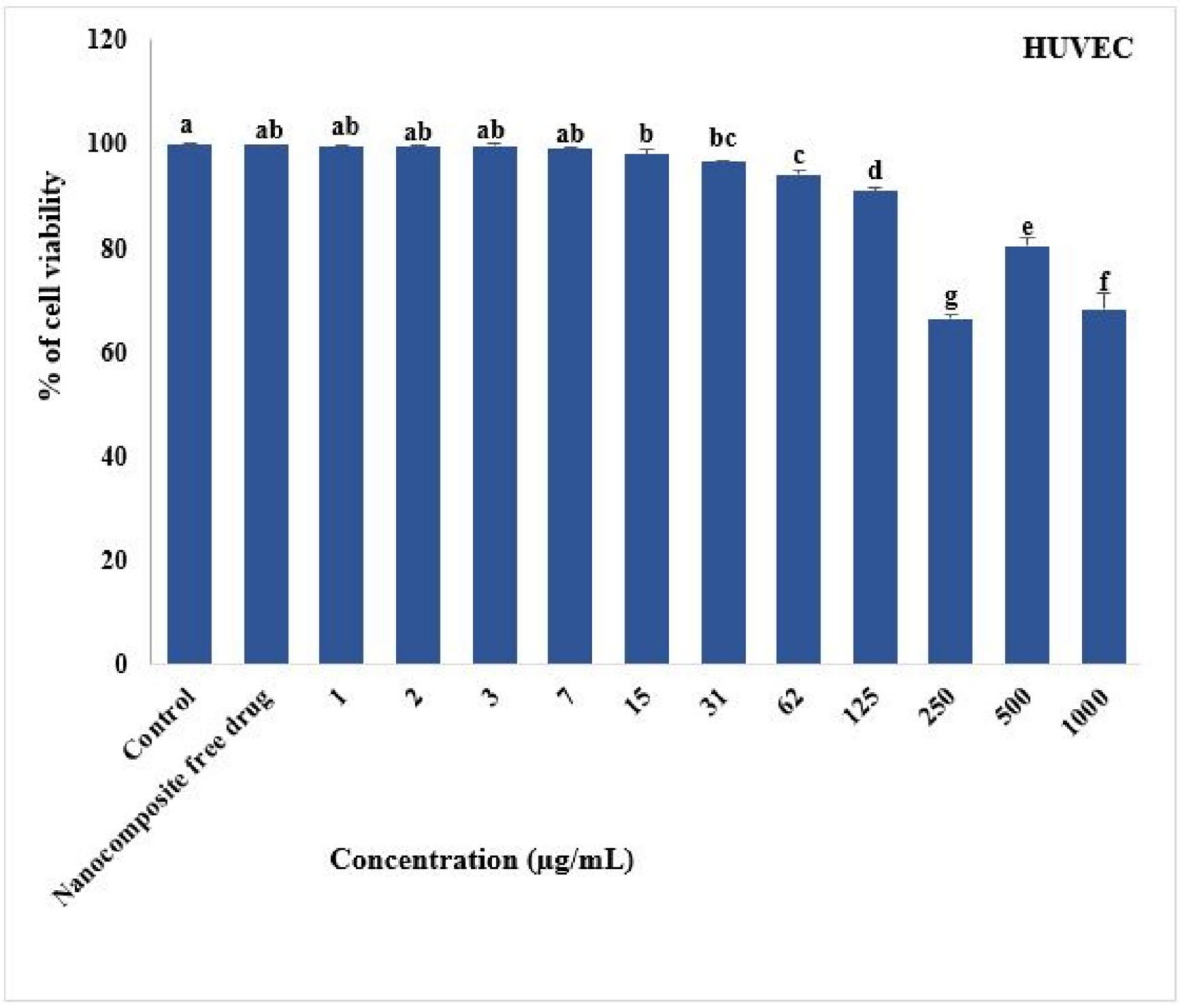
HUVEC cells exhibited a lower level of toxicity.

The cytotoxicity results demonstrated that CNH-LDH@Fe_2_O_3_ beads exhibited low toxicity toward HUVEC cells across the investigated concentration range (Fig. 6). Cell viability remained relatively high even at elevated concentrations, indicating acceptable biocompatibility of the prepared nanocomposite hydrogel system. The data are presented as mean $\:\pm\:$ SD (*n* = 3), and statistical analysis revealed significant differences among some treatment groups (*p* ˂ 0.05).

after exposure to varying concentrations of CNH beads. In addition, the drug-free CNH nanocomposite control exhibited minimal cytotoxicity, suggesting acceptable biocompatibility of the carrier system under the experimental conditions investigated.

### The cytotoxicity of CNH-LDH@Fe_2_O_3_ beads on CML cells

**Fig. 7:**
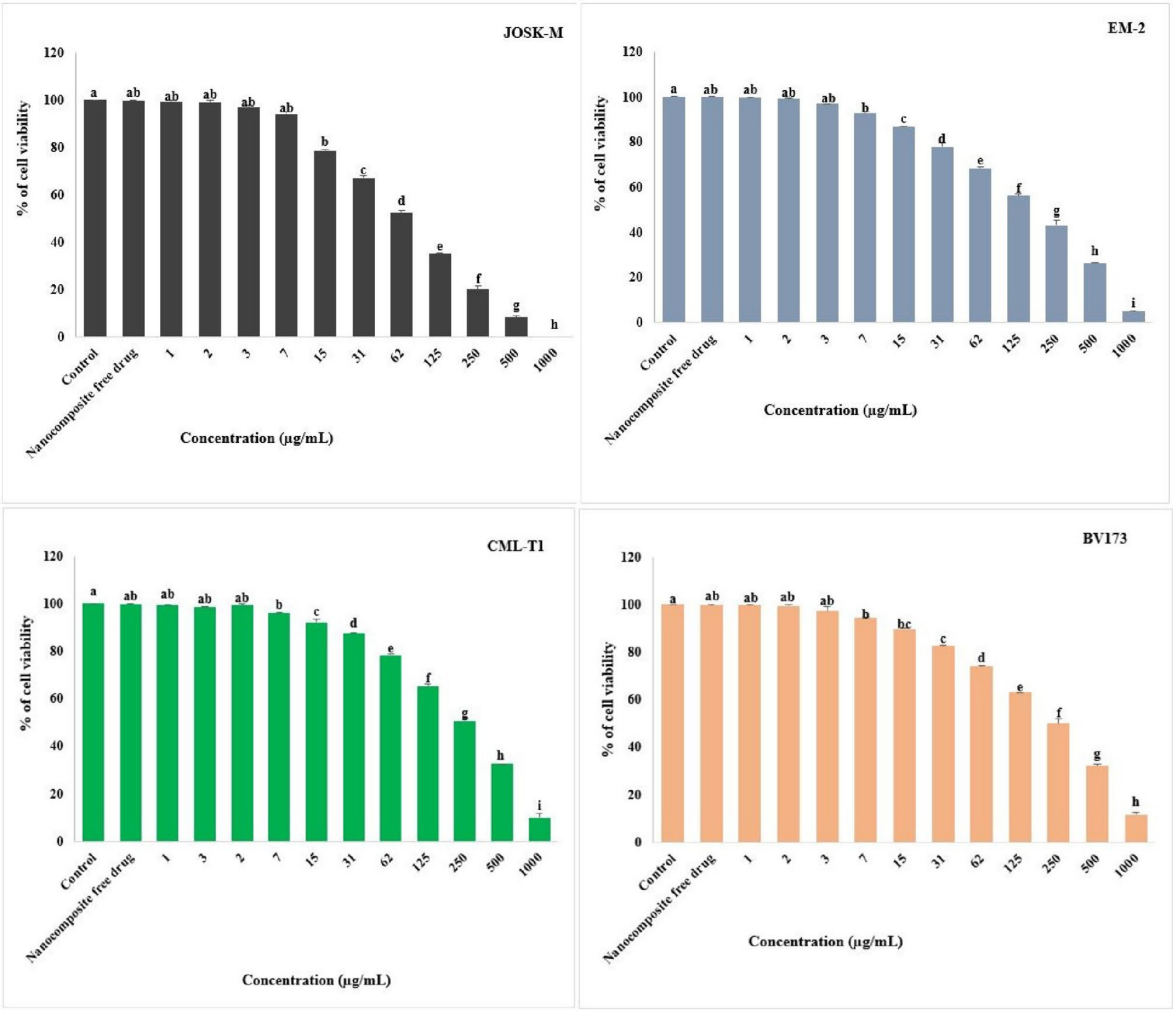
Cytotoxic effects of CNH beads on CML cell lines JOSK-M, EM-2, CML-T1, and BV173. Cytotoxicity evaluation in CML cell lines (JOSK-M, EM-2, CML-T1, and BV173) demonstrated that the investigated CNH-LDH@Fe₂O₃ formulations exhibited concentration-dependent cytotoxicity against leukemic cells.

Cell viability results for CML cell lines demonstrated concentration-dependent cytotoxic effects of the CNH beads against leukemic cells (Fig. 7). Increasing nanocomposite concentration progressively reduced cell viability in the BV173, CML-T1, EM-2, and JOSK-M cell lines. Results are expressed as mean ± SD (*n* = 3). Statistical analysis using Duncan’s multiple range test indicated significant differences among treatment groups at different concentrations (*p* < 0.05).

### Antioxidant effects of CNH-LDH@Fe_2_O_3_ beads

**Fig. 8:**
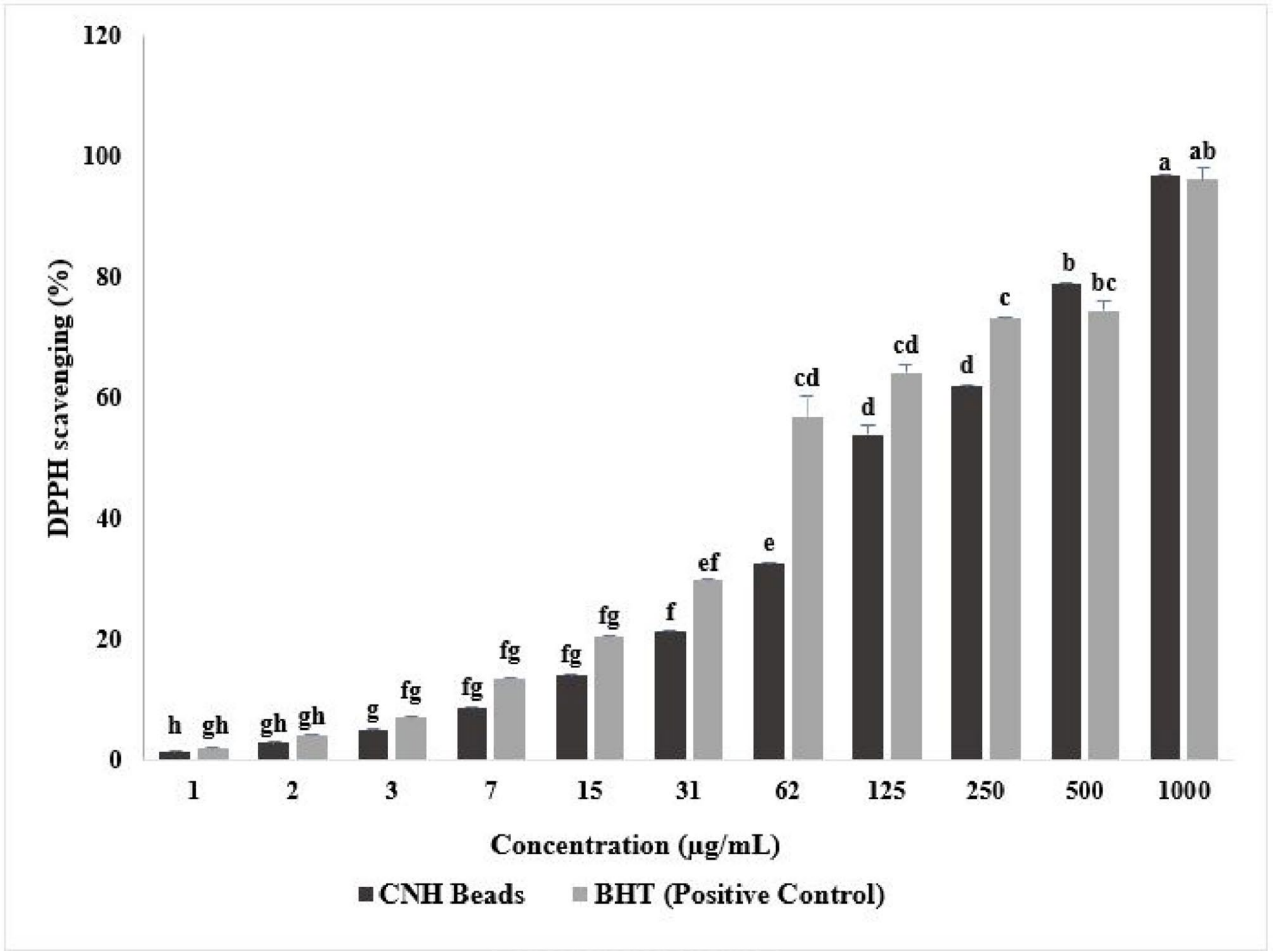
Antioxidant activity of CNH beads and BHT against DPPH radicals.

The antioxidant activity of CNH-LDH@Fe_2_O_3_ beads was evaluated using the DPPH radical-scavenging assay (Fig. 8). The results demonstrated concentration-dependent free radical-scavenging activity of the prepared nanocomposite hydrogel. At higher concentrations, the antioxidant activity of the CNH-LDH@Fe₂O₃ beads approached that of the positive control (BHT), indicating notable antioxidant potential of the synthesized formulation.

## Discussion

Current research in targeted cancer therapy is increasingly concentrating on the development of advanced drug delivery systems, including nanoparticle-based hydrogels, to address the limitations of conventional chemotherapy, such as poor bioavailability, non-specific distribution, and systemic toxicity^33^. In this study, a chitosan-based LDH/Fe₂O₃ nanocomposite hydrogel system was developed to enable controlled delivery of nilotinib for treating CML.

SEM analysis demonstrated that the prepared hydrogels exhibited rough, wrinkled, and porous surface morphologies characteristic of chitosan-based hydrogel matrices^34^. The interconnected porous structure may facilitate drug encapsulation and diffusion within the hydrogel network. In addition, dispersed spherical NP domains observed throughout the matrix confirm the successful incorporation of LDH and LDH/Fe₂O₃ nanocomposites into the hydrogel framework^34^. Increasing NP incorporation ratios further altered the surface roughness and pore distribution of the hydrogels, indicating that NP loading influences the structural organization of the polymeric network. Corresponding morphological features have been documented in previously studied nanoparticle-loaded chitosan hydrogels^29,30^.

XRD analysis further confirmed the successful formation of the Ni–Al-LDH crystalline phase and the incorporation of Fe₂O₃ NPs into the hydrogel matrix. The slight peak shifts and reduced diffraction intensity observed after nanocomposite formation suggest intermolecular interactions among LDH, Fe₂O₃ NPs, and the polymeric hydrogel network^35^. Such interactions may enhance structural homogeneity and stabilize the nanocomposite system, consistent with previous studies on polymer-based nanocomposite hydrogels^36,37^.

In vitro drug-release studies demonstrated that Dry-CNH formulations achieved higher cumulative nilotinib release than Syn-CNH formulations. Notably, Dry-CNH-LDH@Fe₂O₃ 20% exhibited the most significant overall drug release. This enhanced release is presumably attributable to the hydrogel’s porous structure and the influence of Fe₂O₃ nanoparticles on swelling and diffusion within the hydrogel matrix. These findings are consistent with previous research indicating that Dry-CNH can improve release efficiency in hydrogel-based delivery systems^38^.

The antioxidant activity of the CNH-LDH@Fe₂O₃ beads was evaluated using the DPPH radical-scavenging assay. The findings demonstrated a concentration-dependent antioxidant response, with the nanocomposite hydrogel’s radical-scavenging capacity approaching that of the positive control (BHT) at higher concentrations. Although the primary aim of this research was to enable controlled nilotinib delivery, the observed antioxidant activity may be a valuable ancillary characteristic of the developed hydrogel system, especially given the documented role of oxidative stress and ROS imbalance in the progression of leukemia.

The cytotoxicity assessment indicated that the CNH-LDH@Fe₂O₃ hydrogel beads exhibit low toxicity toward normal HUVECs. Conversely, concentration-dependent cytotoxicity was observed in CML cell lines, including BV173, EM-2, CML-T1, and JOSK-M. As the hydrogel concentration increased, HUVEC viability remained relatively high, indicating acceptable biocompatibility of the synthesized nanocomposite system^39^. In contrast, increasing hydrogel concentration significantly reduced leukemic cell viability. Leukemic cells generally exhibit higher metabolic demands and proliferation rates, which may increase their sensitivity to nanocomposite-induced cellular stress. During the experiments, cellular morphology and culture conditions were monitored qualitatively using light microscopy. Concentration-dependent reductions in cell density and cellular shrinkage were observed in CML cell lines, whereas HUVEC cells maintained a comparatively normal morphology. These qualitative observations supported the quantitative viability findings.

Overall, the developed CNH-LDH@Fe₂O₃ hydrogel system demonstrated favorable structural characteristics, controlled nilotinib release, acceptable biocompatibility with normal cells, and selective cytotoxicity against leukemic cell lines. These findings suggest that the prepared nanocomposite hydrogel may represent a promising platform for sustained nilotinib delivery in CML-related applications.

## Conclusions

In this study, CNH-LDH and CNH-LDH@Fe₂O₃ hydrogel beads were successfully developed as chitosan-based nanocomposite systems for controlled nilotinib delivery in CML-related applications. The results demonstrated that incorporating Fe₂O₃ NPs and employing a nilotinib incorporation method influenced the cumulative drug-release profiles of the hydrogel formulations. Furthermore, the CNH-LDH@Fe₂O₃ formulations exhibited concentration-dependent antioxidant activity and selective cytotoxicity against CML cell lines, while maintaining relatively low toxicity toward normal HUVECs. These findings indicate that the developed nanocomposite hydrogel system demonstrates commendable biocompatibility and effective drug-delivery properties. In summary, the prepared hydrogel system has the potential to serve as a promising platform for the sustained release of hydrophobic anticancer agents such as nilotinib. Further in vivo studies are necessary to assess pharmacokinetics, biodegradation behavior, and therapeutic efficacy within physiological conditions.

## Data Availability

The data supporting this study’s findings are available from the corresponding author upon reasonable request.
